# 

**Published:** 1833-07

**Authors:** 


### THE LONDON MEDICAL AND PHYSICAL JOURNAL.

EDITED BY

JOHN NORTH, Esq. F.L.S.

MEMBER OF THE ROYAL COLLEGE OF SURGEONS, AND OF THE MEDICAL AND CHIRURGICAL SOCIETY OF LONDON;

AND

GILBERT T. BURNETT, Esq.

F.L.S. M.B. R.I. R.C.S. &c.

PROFESSOR OF BOTANY IN KING'S COLLEGE, LONDON, AND TO THE MEDICO-BOTANICAL SOCIETY.

(VOL. LXX.)

NEW SERIES, VOL. XV.

Et quoniam variant morbi, variabimus artes;

Mille mali species, mille salutis erunt.

LONDON:

JOHN SOUTER, 73, ST. PAUL'S CHURCH-YARD;

AND TO BE HAD OF ALL MEDICAL BOOKSELLERS.

1833.

J. and C. Adlard, Printers,
Bartholomew Close.

610.5
M 454 v. 70
n. s v. 15

### INDEX

TO THE

FIFTEENTH VOLUME OF THE NEW SERIES

OF

**THE LONDON MEDICAL AND PHYSICAL JOURNAL.**

| | PAGE. |
|---|---|
| ANENCEPHALISM, case of | 163 |
| Apothecaries, Memorial of | 76 |
| their reply to the Edinburgh surgeons | 78 |
| Apothecaries' Hall; names of gentlemen passed | 88, 176, 264 |
| Asthma, review of Dr. Copland's article on | 126 |
| Casper Hauser, curious account of, who was kept in a dungeon for many years | 170 |
| Cerebrum, disease of, illustrative of phrenology | 71 |
| Cholera, autopsy of epidemic | 3 |
| etiology of epidemic | 101, 182 |
| causes of | 192 |
| treatment of | 200 |
| cases of | 244 |
| Dr. Gaulter on, at Manchester | 156 |
| Concours at Paris | 175 |
| Croup, treatment of the first stage of | 74 |
| Crystallization, cause of | 75 |
| Dropsy from feeding on bad potatoes | 164 |
| Empyema, cases of, cured by operation | 212 |
| Encephalon, on the functions of | 255 |
| Fletcher, Mr., notice of his "Sketches from the Case-book | 57 |
| Fœtus found in the mesentery of a boy | 260 |
| Gangrene from feeding on bad potatoes | 164 |
| Gonorrhœa produced by *swallowing* gonorrhœal discharge | 179 |
| Hemeralopia, cases of | 65 |
| Hernia, case of, through the thyroid foramen | 1 |
| Howship, Mr., his Hunterian oration | 75 |
| Hydrocephalus, case of | 61 |
| Hysteria, remarks on bleeding in | 242 |
| Lemons, use of oil of, in ophthalmia | 23 |
| Medical plants mentioned by Shakspeare | 15, 121 |
| Midwifery, Dr. Waller's report on | 117 |
| Mineral waters, notice of Sir C. Scudamore's work on | 243 |
| Navy Board, French certificates recognized at the | 175 |
| Nervous disorders, on | 47 |
| Ophthalmia, oil of lemons used in cases of | 23 |
| Paralysis, strychnine in | 89 |
| Pettigrew, Mr., his work on Mummies announced | 87 |
| Prize essays proposed by the Medical Reform Association | 263 |
| Purpura hemorrhagica, pathology of | 67 |
| Rectum, successful operation for cancer of the | 63 |
| Respiration, inquiry into the causes of | 217 |
| Rot, Professor Faraday on dry, in timber | 150 |
| Sarsaparilla from Malacca | 211 |
| Satyriasis, case of | 58 |
| Strychnine in paralysis | 89 |
| Testicles, observations on | 33 |
| Typhus, review of Dr. Millar on contagious | 142, 231 |
| Urinary abscess, cases of | 159 |
| Urine, influenee of vinegar in lessening the quantity of | 174 |

199597

### INDEX.

### CRITICAL ANALYSES.

Observations on the Testicle. By James Russell, Esq. . . 33
A Treatise on some Nervous Disorders; being chiefly intended to illustrate those Varieties which simulate Structural Disease. By Edwin Lee, Esq. 46
Sketches from the Case-book, to illustrate the Influence of the Mind on the Body. By R. Fletcher, Esq. . 57
Asthma. By Dr. Copland . 126
Clinical Lectures on the Contagious Typhus epidemic in Glasgow and the Vicinity during the Years 1831 and 1832. By Richard Millar, M.D. 142, 231
On the Practical Prevention of Dry Rot in Timber. By Professor Faraday 150
The Origin and Progress of the Malignant Cholera in Manchester. By Henry Gaulter, M.D. . . 156
An Inquiry into the Causes of Respiration; of the Motion of the Blood, Animal Heat, Absorption, and Muscular Motion: with Practical Inferences. By John Carson, M.D. . 216
The Principles and Practice of Obstetric Medicine. By David D. Davis, M.D. . . . 241
A Treatise on the Composition and Medical Properties of the Mineral Waters of Buxton, Matlock, Tunbridge Wells, Harrogate, Bath, Bristol, Cheltenham, Leamington, Malvern, Isle of Wight, Brighton, and the Beulah Spa, Norwood, &c. By Sir Charles Scudamore, M.D. . 243
The Teeth, in relation to Beauty, Voice, and Health. By John Nicholls, Surgeon-Dentist . . 244

### HOSPITAL REPORTS.

Satyriasis, consecutive to a Blow received on the Occipital Region. By M. Chauffard . . 58
Hydrocephalus, with Tubercle and Polypus of the Cerebellum, in a Child of Four Years of Age. By Dr. Guillet . . . 61
Carcinoma of the Rectum; Operation; Recovery . . . 63
Cases of Urinary Abscess . 159
Cases of Catarrhal Cholera. By A. Turnbull Christie, M.D. . 244
Cases of the Mixed Form of Cholera 252
Cases of Inflammatory Cholera . 254

---

COLLECTANEA . . 67, 163, 255

INTELLIGENCE . . 75, 174, 263

METEOROLOGICAL REGISTER 88, 176, 264

---

J. and C. Adlard, Printers, Bartholomew Close.

### MONTHLY LIST OF MEDICAL BOOKS.

[*Medical Works cannot be entered on this List unless a copy be sent for the purpose, the titles of Books having frequently been sent to us as published, whieh have not appeared for weeks, or even months, after.*]

The Cyclopædia of Practical Medicine. Edited by John Forbes, M.D., F.R.S., &c., Alex. Tweedie, M.D., &c., and John Conolly, M.D., &c. Part XVI. Containing, *Paralysis*, (concluded), by Dr. Todd; *Parotitis*, Dr. Kerr; *Pellagra*, Dr. Kerr; *Pemphigus*, Dr. Corrigan; *Perforation of Viscera*, Dr. Carswell; *Pericarditis*, and *Carditis*, Dr. Hope; *Peritonitis*, Dr. Mac Adam and Dr. Stokes; *Persons found Dead*, Dr. Beatty; *Phlegmasia Dolens*, Dr. Lee; *Pityriasis*, Dr. Cumin; *Plague*, Dr. Brown; *Plethora*, Dr. Barlow.—Large 8vo. pp.127. London: Sherwood, Gilbert, and Piper.

The Origin and Progress of the Malignant Cholera in Manchester, considered chiefly in their bearing on the Contagiousness and the Secondary Causes of the Disease. To which are added, some Remarks on the Treatment. With an illustrative Chart. By Henry Gaulter, M.D., of Magdalen College, Oxford, &c.—8vo. pp. 206. Longman, London.

Part XX. The Principles and Practice of Obstetric Medicine, in a Series of Systematic Dissertations on Midwifery and on the Diseases of Women and Children. Illustrated by numerous Plates. By D. D. Davis, M.D., &c.—4to. John Taylor, London.

Sketches from the Case-book, to illustrate the Influence of the Mind on the Body; with the Treatment of some of the more important Brain and Nervous Disturbances which arise from this Influence. By R. Fletcher, Esq., Surgeon to the Glocester General Hospital, &c.—8vo. pp. 391. London: Longman and Co.; Simpkin and Marshall.

Clinical Lectures on the Contagious Typhus, epidemic in Glasgow and the Vicinity, during the Years 1831 and 1832. By Richard Millar, M.D., Senior Physician to the Royal Infirmary, &c.—8vo. pp. 144. London: Longman and Co.

A Treatise on some Nervous Disorders; being chiefly intended to illustrate those Varieties which simulate Structural Disease. By Edwin Lee, Member of the Royal College of Surgeons; formerly House Surgeon to St. George's Hospital.—8vo. pp. 152. London: Burgess and Hill.

### APOTHECARIES' HALL.

**Names of Gentlemen** to each of whom the Court of Examiners have granted Certificates:

**May 30.**

John S. Burgess Budgett, Shepton Mallet
Edward Drewry, Derby
George Dunn, Barnsley
Henry Patrick Finiccaine, Dublin
James Joseph Power
John Steele, Borden, Kent
Henry Somervillé, Stafford
James Bailey Toldervey, Croydon

**June 6.**

Henry Bell, Leeds
William Murdoch, Rotherhithe
Edward Young, Hawkhurst

**June 13.**

William Atkinson
William Ball, Todwick
Thomas Butterworth, Manchester
Robert Cartwright, Oswestry
Charles Dyer, London
Thomas Nicholas Heygate, Hanslope
Frederick Gilder Julius, Richmond
John Peck Margetts, Huntingdonshire
Robert Knewstubb Richmond, Chatham
Frederick Charles Wright, London

**June 20.**

John Brown
Frederick Josiah Burgess
Edwin Unwin Berry.

